# Branched-Chain Fatty Acids as Mediators of the Activation of Hepatic Peroxisome Proliferator-Activated Receptor Alpha by a Fungal Lipid Extract

**DOI:** 10.3390/biom10091259

**Published:** 2020-08-31

**Authors:** Garima Maheshwari, Robert Ringseis, Gaiping Wen, Denise K. Gessner, Johanna Rost, Marco A. Fraatz, Holger Zorn, Klaus Eder

**Affiliations:** 1 Institute of Animal Nutrition and Nutrition Physiology, Justus Liebig University Giessen, Heinrich-Buff-Ring 26-32, 35392 Giessen, Germany; Garima.Maheshwari@lcb.chemie.uni-giessen.de (G.M.); gaiping.wen@ernaehrung.uni-giessen.de (G.W.); denise.gessner@ernaehrung.uni-giessen.de (D.K.G.); Klaus.Eder@ernaehrung.uni-giessen.de (K.E.); 2 Institute of Food Chemistry and Food Biotechnology, Justus Liebig University Giessen, Heinrich-Buff-Ring 17, 35392 Giessen, Germany; johanna.rost@gmx.de (J.R.); marco.fraatz@lcb.chemie.uni-giessen.de (M.A.F.); holger.zorn@lcb.chemie.uni-giessen.de (H.Z.); 3 Fraunhofer Institute for Molecular Biology and Applied Ecology, Winchester Str. 2, 35394 Giessen, Germany

**Keywords:** branched-chain fatty acids, *Conidiobolus heterosporus*, peroxisome proliferator-activated receptor α, lipid metabolism, fatty acid oxidation, hepatocyte

## Abstract

The study aimed to test the hypothesis that monomethyl branched-chain fatty acids (BCFAs) and a lipid extract of *Conidiobolus heterosporus* (CHLE), rich in monomethyl BCFAs, are able to activate the nuclear transcription factor peroxisome proliferator-activated receptor alpha (PPARalpha). Rat Fao cells were incubated with the monomethyl BCFAs 12-methyltridecanoic acid (MTriA), 12-methyltetradecanoic acid (MTA), isopalmitic acid (IPA) and 14-methylhexadecanoic acid (MHD), and the direct activation of PPARalpha was evaluated by reporter gene assay using a PPARalpha responsive reporter gene. Furthermore, Fao cells were incubated with different concentrations of the CHLE and PPARalpha activation was also evaluated by using the reporter gene assay, and by determining the mRNA concentrations of selected PPARalpha target genes by real-time RT-PCR. The reporter gene assay revealed that IPA and the CHLE, but not MTriA, MHD and MTA, activate the PPARalpha responsive reporter gene. CHLE dose-dependently increased mRNA concentrations of the PPARalpha target genes acyl-CoA oxidase (ACOX1), cytochrome P450 4A1 (CYP4A1), carnitine palmitoyltransferase 1A (CPT1A) and solute carrier family 22 (organic cation/carnitine transporter), member 5 (SLC22A5). In conclusion, the monomethyl BCFA IPA is a potent PPARalpha activator. CHLE activates PPARalpha-dependent gene expression in Fao cells, an effect that is possibly mediated by IPA.

## 1. Introduction

The peroxisome proliferator-activated receptor alpha (PPARalpha) is a transcription factor that belongs to the superfamily of nuclear hormone receptors and is predominantly expressed in tissues with high rates of fatty acid oxidation. PPARalpha can be activated by micromolar concentrations of a variety of peroxisome proliferators including synthetic agonists, such as fibrates, a class of hypolipidemic drugs, and natural agonists, such as fatty acids (e.g., dietary ω-3 polyunsaturated fatty acids (PUFAs), like arachidonic acid (AA), linoleic acid (LA) or α-linolenic acid (ALA)), eicosanoids and their derivatives [1,2,3]. Upon activation, PPARalpha upregulates the expression of a large set of target genes involved in fatty acid uptake, transport and oxidation through binding to a specific sequence, called peroxisome proliferator response element (PPRE), in the regulatory region of these genes [4]. In addition, PPARalpha inhibits several proinflammatory genes through a negative crosstalk with the key regulator of inflammation, nuclear factor-kappa B, especially in the vasculature [5]. Owing to this, PPARalpha activators exhibit lipid-lowering and atheroprotective effects, thereby reducing the risk of developing cardiovascular diseases (CVDs) [6]. 

Apart from the abovementioned PPARalpha activators, the tetramethyl branched-chain, isoprenoid-derived fatty acids phytanic acid (3,7,11,15-tetramethylhexadecanoic acid) and pristanic acid (2,6,10,14-tetramethylpentadecanoic acid) as well as their CoA-thioesters were shown to be high-affinity natural ligands and potent activators of PPARalpha [7,8,9,10]. In addition, the feeding of phytol, which is metabolized to phytanic acid following absorption from the intestine, was found to activate PPARalpha in tissues of mice [11]. Branched-chain fatty acids (BCFAs) are primarily saturated fatty acids, both even-carbon- and odd-carbon-numbered, with one or more methyl branches, accordingly called mono- or multimethyl BCFAs, respectively. The branching is mainly at the penultimate (*iso*) or next to the penultimate carbon atom (*anteiso*). BCFAs belong to the minor fatty acids in food and are found in ruminant products (dairy and beef) due to synthesis by ruminal microorganisms [12,13]; they are also present at significant levels in fermented foods of animal (shrimp paste, fish sauce) and nonanimal origin (sauerkraut) [13,14]. In addition, BCFAs occur naturally in bacterial lipids and in several fungi [15,16]. As early as 1967, Tyrrell described the composition of fatty acids in various species of the fungal genus *Conidiobolus* and reported proportions of BCFAs of up to 73% of total fatty acids [17]. Among these *Conidiobolus* species, the *Conidiobolus heterosporus* Drechsler 1953, first described by Tyrrell in 1971, belonging to the phylum *Zygomycota*, contains 53% of the total fatty acids as BCFAs, with the main BCFA being 12-methyltridecanoic acid (MTriA, *iso*-C14:0) (33% of total fatty acids), 12-methyltetradecanoic acid (MTA, *anteiso*-C15:0) (13%), 14-methylpentadecanoic acid (=isopalmitic acid, IPA, *iso*-C16:0) (6%) and 14-methylhexadecanoic acid (MHD, *anteiso*-C17:0) (1%) [18]. In contrast to the multimethyl BCFAs phytanic acid or pristanic acid, the BCFA species contained in *Conidiobolus heterosporus*, like MTriA, MTA, IPA and MHD, are monomethyl BCFAs. Whether these monomethyl BCFAs are also able to activate PPARalpha, however, is currently unknown.

Therefore, the present study aimed to test the following two hypotheses: First, based on the structural similarity between multimethyl BCFAs and monomethyl BCFAs, we tested the hypothesis that monomethyl BCFAs are potent PPARalpha activators. Second, due to the high amount of monomethyl BCFAs in *Conidiobolus heterosporus* lipids, we tested the hypothesis that a lipid extract of *Conidiobolus heterosporus* (CHLE) also activates PPARalpha and thereby induces the expression of PPARalpha target genes. To test these two hypotheses, we treated rat Fao hepatoma cells, a frequently used cell line to investigate the ability of different substances to activate the PPARalpha pathway, with the monomethyl BCFAs MTriA, MTA, IPA and MHD, in isolated form and with various concentrations of the CHLE and tested their ability to activate PPARalpha by determining PPARalpha transactivation and induction of PPARalpha target genes. As a positive control, we used WY-14643, a synthetic agonist of PPARalpha, and two PUFAs, LA and ALA, both of which are naturally occurring PPARalpha agonists [2].

## 2. Materials and Methods

### 2.1. Submerged Cultivation of Conidiobolus heterosporus, Fungal Lipid Extraction and Preparation of Fatty Acid Methyl Esters

*Conidiobolus heterosporus* Drechsler 1953, which belongs to the phylum Zygomycota, order Entomophtorales, family Ancylistaceae and the genus *Conidiobolus*, was obtained from Centraalbureau voor Schimmelcultures (CBS 138.57; Utrecht, Netherlands), and grown in submerged cultures following a previously described method [19]. For the precultures, 100 mL of yeast extract (3 g/L) and malt extract (30 g/L) (YM) medium (Merck KGaA, Darmstadt, Germany) in 250 mL Erlenmeyer flasks was used. After 3 days, the precultures were homogenized for 30 s at 9800 rpm using an Ultra Turrax homogenizer (IKA, Staufen, Germany). For the main cultures, 20 mL of the homogenized suspension was used to inoculate 200 mL of YM medium in 500 mL Erlenmeyer flasks. The cultivations were carried out in an Ecotron incubation shaker (25 mm shaking diameter, 150 rpm, 24 °C) (Infors GmbH, Einsbach, Germany) for 5 days. For harvesting, the fungal mycelium was separated from the culture broth by vacuum filtration with a Buchner funnel (110 mm) and a DP 595 cellulose filter paper (Albet LabScience, Dassel, Germany).

Total lipids were extracted by the Soxhlet method. After saponification of 150 mg CHLE with 4 mL 0.5 M NaOH in methanol (80 °C, 10 min), free fatty acids were converted into their corresponding fatty acid methyl esters (FAMEs) by addition of 3.5 mL boron trifluoride-methanol solution (20%) and heating to 80 °C for 5 min as described previously [19]. After addition of 1 mL n-hexane and incubation at 80 °C for 1 min, 3 mL saturated NaCl solution was added. The organic phase was dried over anhydrous sodium sulfate overnight.

### 2.2. Analysis of Fatty Acid Composition

The fatty acid composition of the fungal lipid extract was determined by using an Agilent 7890A gas chromatograph (Agilent Technologies, Waldbronn, Germany), equipped with a model 5975C mass spectrometry detector (Agilent Technologies), under the following conditions: carrier gas, helium (5.0); constant flow rate, 1.2 mL/min; inlet temperature, 250 °C; split ratio, 10:1; septum purge flow rate, 3 mL/min; 30 m × 0.25 mm i.d., 0.25 μm BP21 (FFAP) column (SGE Europe Ltd, Milton Keynes, UK); temperature program, 40 °C (3 min) and 5 °C/min to 240°C (12 min); scan mode, TIC; scan range, m/z 33–400; electron ionization energy, 70 eV; source temperature, 230 °C; quadrupole temperature, 150 °C; transfer line temperature, 250 °C. The identification of the fatty acids was carried out by the comparison of retention indices with the reference standard Supelco 37 component FAME mix (Sigma-Aldrich, Taufkirchen, Germany). To determine the fatty acid composition, all FAME peak areas were summed up, set to 100%, and the results were expressed as a relative percentage for each fatty acid. The fatty acid composition of the CHLE is listed in Table 1.

### 2.3. Chemical Reagents

Ham’s F12 medium, fetal calf serum (FCS), gentamycin and Trizol were purchased from Invitrogen (Karlsruhe, Germany). WY-14,643; the isolated fatty acids LA (≥99% pure), ALA (≥99% pure), IPA (≥98% pure), MTriA (≥98% pure), MHD (≥98% pure) and MTA (≥98% pure); and the MTT (3-(4,5-dimethylthiazole-2-yl)-2,5-diphenyltetrazolium bromide; Thiazole Blue) stock solution were obtained from Sigma-Aldrich (Steinheim, Germany).

### 2.4. Cell Culture

The rat hepatoma Fao cell line [20] was obtained from the European Collection of Cell Cultures (ECACC Cat. No. 89042701; Salisbury, UK) and grown in Ham’s F12 medium supplemented with 10% FCS and 0.5% gentamycin at 37 °C and humidified atmosphere of 95% air and 5% CO_2_. Fao cells were seeded either in 24-well culture plates (Greiner Bio-One, Frickenhausen, Germany) at a cell density of 2.1 × 10^5^ per well for cell viability assay and qPCR analysis or in 96-well culture plates at a cell density of 17 × 10^4^ per well.

### 2.5. Cell Treatments

After reaching 70–80% confluency, cells were treated with either CHLE, WY-14,643 or various isolated fatty acids (LA, ALA, MTriA, MTA, IPA or MHD) for 24 h at the concentrations indicated. Incubation media containing isolated fatty acids were prepared by diluting the fatty acid stock solutions (100 mM in ethanol) with low-serum Ham’s F12 medium (0.5% FCS) as described previously [21]. Prior to adding CHLE into the incubation media, an aliquot of the stock solution (100 mM in ethanol, based on the molecular weight of an average triglyceride of 850) was evaporated under a nitrogen stream and dissolved in 0.03 N NaOH at room temperature to saponify triglycerides and bidistilled water. Afterwards, the saponified CHLE was diluted in low-serum Ham’s F12 medium (0.5% FCS) (pH 7-8). WY-14,643 was added to the low-serum medium from a 100 mM stock solution dissolved in DMSO. Cells treated with the vehicle alone (DMSO for WY-14,643, ethanol for isolated fatty acids, low-serum medium for CHLE) were used as controls and contained the same vehicle (ethanol) concentration (ethanol: 0.5 % (*v*/*v*); DMSO: 0.05% (*v*/*v*)). After addition of either CHLE, isolated fatty acids or WY-14,643 to the medium, the medium was gently mixed at RT to ensure complete solubility of the added substances. No signs of precipitation could be observed.

### 2.6. Cell Viability Assay

Cell viability after treatment with either CHLE or isolated fatty acids was assessed by the MTT assay [22]. Briefly, after removing the incubation media, 5 mg/mL MTT stock solution dissolved in PBS (140 mM NaCl, 3 mM KCl, 8 mM Na_2_HPO_4_, 1.8 mM KH_2_PO_4_; pH 7.4) was added to each well, and cells were incubated at 37 °C for 4 h. Subsequently, the MTT/PBS solution was removed, and the formazan crystals generated during the incubation period were dissolved by adding isopropanol in 0.04 N HCl. After the crystals were completely dissolved, the solution was transferred into a 24-well plate, and its absorbance was measured at the wavelength of 540 nm using an Infinite 200 M microplate reader (Tecan, Männedorf, Switzerland). Cell viabilities are expressed as percentage of control cells, which was set to 100%.

### 2.7. Transient Transfection and Dual Luciferase Reporter Assay

After reaching 70–80% confluency, cells were transiently transfected with 50 ng of a 3X ACO-PPRE vector (containing three copies of consensus PPRE from the ACO promoter in front of a luciferase reporter gene) and pGL4.10 vector as negative control using FuGENE 6 transfection reagent (Roche diagnostics, Mannheim, Germany) for 12 h according to the manufacturer’s protocol. Cells were also co-transfected with 5 ng of pGL4.74 Renilla luciferase (encoding the Renilla luciferase reporter gene; Promega, Mannheim, Germany), which was used as internal control reporter vector to normalize for differences in transfection efficiency (Promega). After transfection, cells were treated with either WY-14643; the isolated fatty acids LA, ALA, MTriA, MTA, IPA or MHD; or the CHLE at the concentrations indicated or vehicle alone for 24 h. Afterwards, cells were washed with PBS and lysed with lysis buffer (Promega). Luciferase activities were determined with Beetle-Juice and Renilla-Juice Kits from PJK (Kleinblittersdorf, Germany) in a Mithras LB940 luminometer (Berthold Technologies, Bad Wildbad, Germany). For control of background luminescence, Firefly and Renilla luciferase activities were also determined in the lysates of nontransfected control cells and subtracted from luminescence of transfected cells. Data were normalized for transfection efficiency by dividing Firefly luciferase activity of the 3X ACO-PPRE plasmid by that of Renilla luciferase activity of the co-transfected pGL4.74 Renilla luciferase plasmid. Results represent normalized luciferase activities and are shown relative to cells transfected with pGL4.10 vector and treated with the vehicle only, which was set to 1.

### 2.8. Quantitative Real-Time Reverse-Transcription Polymerase Chain Reaction (qPCR)

After treatment, the media were discarded, the cells were washed once with PBS and total RNA was isolated using Trizol reagent according to the manufacturer’s protocol. Total RNA (1.2 µg) was reverse-transcribed using 60 U M-MuLV reverse transcriptase in a Biometra Thermal Cycler (Whatman Biometra, Göttingen, Germany). The cDNA was stored in aliquots at −20 °C. Subsequent real-time RT-PCR analysis of selected PPARalpha target genes and reference genes and calculation of gene expression data was performed as described recently in detail [23]. The normalization factor was calculated as the geometric mean of expression data of the three most stable out of six tested potential reference genes (CANX, MDH1, ACTB, RPL13, TOP1 and ATP5B). Means and SD were calculated from normalized expression data for cells of the same treatment group. The mean of the vehicle control cells was set to 1, and the mean and SD of the treated cells were scaled proportionally. Features of gene-specific primer pairs are listed in Supplementary Table S1.

### 2.9. Statistics

Statistical analysis was performed using the Minitab statistical software (Release 13, Minitab Inc., State College, PA, USA). Normal distribution of variables was tested with the Anderson–Darling test. Since all data showed normal distribution, the effects of different concentrations of CHLE, isolated fatty acids and WY-14643 in comparison to control treatment were analyzed by Student’s *t*-test. Post-hoc analysis was performed using Fisher’s multiple comparison test. Means were considered significantly different from control at *p* ˂ 0.05. Data are means ± SD calculated from three independent experiments. In each independent experiment, all treatments were represented in 4 (qPCR) and 8 (reporter assay, MTT assay) wells (representing the numbers of technical replicates per treatment).

## 3. Results

### 3.1. Effects of Isolated Fatty Acids and CHLE on Cell Viability of Rat Fao Cells

**Figure 1:**
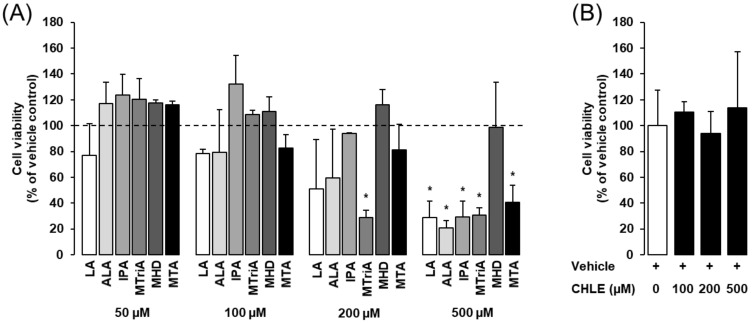
Effects of isolated fatty acids (**A**) and *Conidiobolus heterosporus* lipid extract (CHLE) (**B**) on cell viability of Fao cells. Fao cells were treated either without (vehicle) or with different concentrations of linoleic acid (LA), α-linolenic acid (ALA), isopalmitic acid (IPA), 12-methyltridecanoic acid (MTriA), 14-methylhexadecanoic acid (MHD), 12-methyltetradecanoic acid (MTA) or CHLE for 24 h, and cell viability was measured by the MTT assay. Bars represent means ± SD of three independent experiments. (**A**) The dashed line indicates the vehicle control (=100%). * *p* ˂ 0.05 compared with vehicle control.

Incubation with either isolated BCFAs or straight-chain fatty acids for 24 h did not impair cell viability up to a concentration of about 200 µM, with the exception of MTriA, as demonstrated by the MTT assay (Figure 1A). Cell viability of Fao cells was, however, not reduced by 24 h incubation with the monomethyl BCFA MHD up a concentration 500 µM, the highest concentration tested (Figure 1A). Cell viability of Fao cells was not reduced by 24 h incubation with CHLE up to a concentration of 500 µM (Figure 1B)

### 3.2. Effects of Isolated Fatty Acids and CHLE on PPARalpha Transactivation

**Figure 2:**
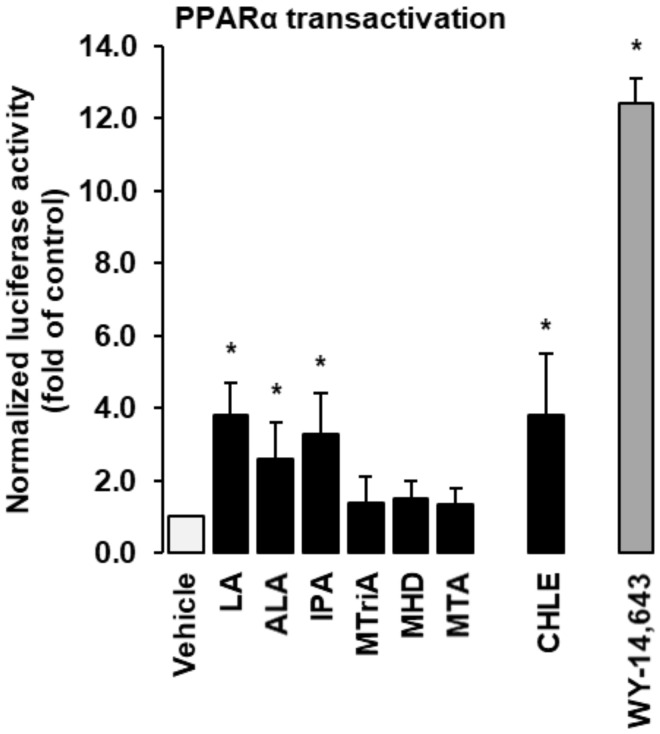
Effects of isolated fatty acids, *Conidiobolus heterosporus* lipid extract (CHLE) and synthetic PPARalpha agonist WY-14,643 on PPARalpha transactivation in Fao cells. Fao cells were transiently transfected with a 3X ACO-PPRE vector and a Renilla luciferase expression vector for normalization using FuGENE6. After transfection, cells were treated either without (vehicle) or with different isolated fatty acids (50 µM each), CHLE (500 µM) or WY-14,643 (50 µM) for 24 h. Afterwards, cells were lysed, and luciferase activities were determined by dual luciferase assay. Bars represent means ± SD of three independent experiments. * *p* ˂ 0.05 compared with vehicle control.

Using a PPARalpha-responsive reporter gene, we studied the effect of CHLE and isolated fatty acids contained in the CHLE on PPARalpha transactivation. As shown in Figure 2, the synthetic PPARalpha agonist WY-14,643 markedly increased the luciferase activity of the PPARalpha-responsive reporter gene about 12-fold compared to vehicle control (*p* < 0.05). IPA, one of the main monomethyl BCFAs of the CHLE, increased the luciferase activity of the reporter gene about 3.5-fold (*p* < 0.05), while the other monomethyl BCFAs, namely MTriA, MHD and MTA, did not (Figure 2). Treatment of cells with CHLE increased the luciferase activity of the PPARalpha-responsive reporter gene about 4-fold compared to vehicle control (*p* < 0.05). The straight-chain fatty acids LA and ALA increased the luciferase activity of the reporter gene about 4- and 3-fold, respectively, compared to vehicle control (*p* < 0.05, Figure 2).

### 3.3. Effect of CHLE on the mRNA Concentration of PPARalpha Target Genes

**Figure 3:**
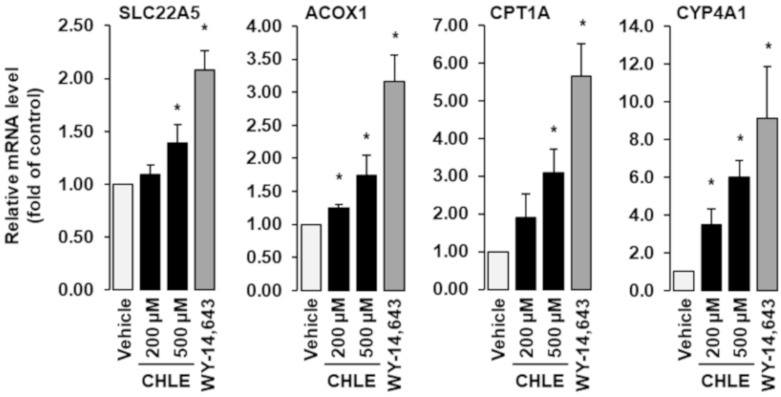
Effects of *Conidiobolus heterosporus* lipid extract (CHLE) on relative mRNA levels of the PPARalpha target genes SLC22A5, ACOX1, CPT1A and Cyp4A1 in Fao cells. Fao cells were treated either without (vehicle) or with CHLE at two different concentrations (200 and 500 µM) or WY-14,643 (50 µM) for 24 h, and mRNA levels were determined by qPCR. Bars represent means ± SD of three independent experiments. * *p* ˂ 0.05 compared with vehicle control.

Treatment with CHLE increased the mRNA concentrations of the PPARalpha target genes solute carrier family 22 (organic cation/carnitine transporter), member 5 (SLC22A5), acyl-CoA oxidase (ACOX1), carnitine palmitoyltransferase 1A (CPT1A) and cytochrome P450 4A1 (CYP4A1) in a dose-dependent manner (*p* ˂ 0.05, Figure 3). The strongest increase was observed for CYP4A1, which was increased 6-fold by 500 µM CHLE compared to control (*p* ˂ 0.05, Figure 3). As expected, the synthetic PPARalpha agonist WY-14,643 caused a markedly stronger induction of all PPARalpha target genes investigated when compared to CHLE (*p* ˂ 0.05, Figure 3).

## 4. Discussion

In the present study, we tested the hypothesis that both isolated monomethyl BCFAs and CHLE, which is rich in monomethyl BCFAs, activate the nuclear transcription factor PPARalpha and CHLE induces the expression of PPARalpha target genes in rat Fao cells. Our data from the reporter gene assay clearly show that the monomethyl BCFA IPA and the CHLE are potent activators of PPARalpha. Moreover, we found that CHLE induces the expression of known PPARalpha target genes involved in fatty acid metabolism, like ACOX1, CPT1A, CYP4A1 and SLC22A5, in a dose-dependent manner. Compared with WY-14,643 the extent of activation of PPARalpha by IPA and CHLE was lower, but this is not surprising given that WY-14,643 is a high-affinity synthetic ligand of PPARalpha and such ligands typically cause a stronger stimulation of PPARalpha transactivation than naturally occurring ligands [2].

Several studies are available reporting that tetramethyl BCFAs, like phytanic acid, pristanic acid and their CoA-thioesters, as well as the synthetically produced short-chain BCFA valproic acid, cause PPARalpha activation [9,24,25]. However, to our knowledge, there is no study available exploring the effect of the monomethyl BCFAs MTriA, MTA, IPA and MHD on PPARalpha activation in isolated form. Thus, our study shows for the first time that the monomethyl BCFA IPA, which makes up about 6% of total fatty acids of CHLE, significantly stimulates PPARalpha transactivation, indicating that IPA is responsible for the effect of the CHLE. A few studies are available investigating the effect of a mixture of different BCFAs in laboratory animals. For instance, Shirouchi et al. [26] showed that feeding 5% porpoise oil, which contains 15.5% BCFAs (including the monomethyl BCFA IPA), to obese rats alleviates hepatic triglyceride accumulation and increases serum adiponectin levels, effects that are known to be mediated by PPARalpha activation [27]. The decrease of hepatic triglyceride accumulation in response to PPARalpha activation is mechanistically explained by the physiological function of the proteins encoded by many PPARalpha target genes, including those considered in the present study as indirect markers of PPARalpha activation. The enzymes encoded by ACOX1, CPT1A and CYP4A1 are directly involved in peroxisomal, mitochondrial and microsomal fatty oxidation pathways, respectively, and induction of these genes causes a decrease of hepatic triglyceride levels due to an increased oxidation of fatty acids [4]. SLC22A5 encodes the novel organic cation transporter OCTN2, which mediates carnitine uptake into tissues. Because carnitine is essential for the transport of long-chain fatty acids into the mitochondrial matrix, where mitochondrial fatty acid oxidation takes place, genetic defects of this carnitine transporter result in decreased intracellular carnitine levels and an impaired fatty acid oxidation [28]. A limitation of the abovementioned study [26] is that porpoise oil also contains ω-3 PUFAs, which are also known to be activators of PPARalpha. Thus, the contribution of BCFAs to the effect of porpoise oil in this study remains unclear. In another study, it was reported that rat pups receiving milk substituted with BCFAs (including IPA) showed an increased intestinal expression of the anti-inflammatory interleukin-10 (IL-10) compared with pups receiving no BCFAs and a reduced incidence of necrotizing enterocolitis [29], which is indicative of an anti-inflammatory action of BCFAs in the intestine. This effect might be also mediated by activation of PPARalpha by the BCFAs, because upregulation of IL-10 has been reported to occur in response to administration of WY-14,643 [30]. In addition, the anti-inflammatory effect of BCFAs in the neonatal rat intestine might be also explained by the well-known negative crosstalk of PPARalpha with critical inflammatory signaling pathways such as nuclear factor-kappa B [5].

In summary, the observation of the present in vitro study that the monomethyl BCFA IPA is an activator of PPARalpha may provide an explanation for the PPARalpha-mediated effects of BCFA-rich diets in in vivo studies [26,29]. Nevertheless, other fatty acids, particularly PUFAs like LA, AA and ALA (all of which were present in the CHLE), are naturally occurring PPARalpha activators [1,2]. Thus, not only LA and ALA, used as further positive controls in the reporter gene assay, but also AA may have also contributed to the PPARalpha-dependent effects of CHLE or BCFA-rich diets. In any case, the results of the present study suggest that dietary supplementation of CHLE might be a useful approach to induce the beneficial effects associated with PPARalpha activation like lipid lowering and reduction of CVD risk.

Considerations with regard to the use of BCFA-rich sources as a dietary supplement have to include the induction of possible adverse effects. In this context, it should be noted that various feeding studies in rats, lambs and steers did not observe any toxic or adverse effects after treatment with different BCFAs [31,32,33]. In contrast, adverse effects including teratogenic, hepatotoxic, neurotoxic and proapoptotic effects have been reported for valproic acid, which is also used as an antiepileptic drug, but only at extremely high concentrations [34,35]. Such effects might be also, at least partially, explained by the activation of PPARalpha, because administration of synthetic PPARalpha activators to rodents was found to cause hepatic peroxisome proliferation, hypertrophy, hyperplasia and even hepatocarcinogenesis [36], with this last condition being attributed to induction of oxidative stress and an imbalance between apoptosis and cell proliferation [37]. Apart from this, clinical observations in patients with peroxisomal disorders like Zellweger syndrome or Refsum’s disease and studies employing corresponding mouse models have shown that elevated concentrations of BCFAs are associated with a high level of toxicity [38,39]. However, it has to be considered that in such cases the BCFA concentrations in blood markedly exceed those achieved by dietary supplementation. Proapoptotic effects were also found for naturally occurring BCFAs, like MTA [40,41]. Such proapoptotic effects however were found to occur in tumor cells, an effect that is probably beneficial. Collectively, it can be stated that more studies are required for the evaluation of safety aspects of BCFA-rich sources as food supplements.

## 5. Conclusions

This study shows for the first time that the monomethyl BCFA IPA and the CHLE are able to induce PPARalpha activation in cultured hepatocytes. These findings indicate that lipid extracts from fungi, such as CHLE, are a source of PPARalpha-activating biomolecules which might be considered as dietary supplements to induce beneficial effects associated with PPARalpha activation, like lipid lowering and reduction of CVD risk, provided that no safety concerns exist. In light of recent evidence showing that administration of BCFAs in neonatal rats increases the expression of anti-inflammatory cytokines and reduces the incidence of necrotizing enterocolitis, an anti-inflammatory action of BCFAs in the intestine might be postulated, which is likely mediated by the well-established negative crosstalk of PPARalpha activation with critical inflammatory signaling pathways. Regarding adverse effects reported from the use of synthetic PPARalpha activators in rodents, potential safety issues associated with BCFA-rich sources as food supplements have to be thoroughly evaluated.

## Figures and Tables

**Table 1:** Fatty acid composition of the *Conidiobolus heterosporus* lipid extract.

Fatty acids ^1^	Area (%)
* Branched-chain fatty acids (BCFAs)*	*52.7*
*iso*-C14:0 (MTriA)	33.0
*anteiso*-C15:0 (MTA)	13.1
*iso*-C16:0 (IPA)	5.9
*anteiso*-C17:0 (MHD)	0.7
* Straight-chain fatty acids*	*39.5*
C12:0	0.3
C13:0	1.4
C14:0	5.4
C14:1	0.1
C15:0	4.8
C16:0	7.8
C16:1	0.6
C17:0	0.4
C18:0	1.3
C18:1	1.7
C18:2	0.7
C18:3 (γ-linolenic acid)	0.8
C20:0	0.2
C20:1	0.2
C20:2	0.3
C20:3	1.1
C20:4	9.4
C20:5	0.4
C22:0	0.2
C22:1	0.3
C22:2	0.3
C23:0	0.1
C24:0	1.4
C24:1	0.3
*Sum of saturated straight-chain fatty acids*	*23.3*
*Sum of monounsaturated straight-chain fatty acids*	*3.2*
*Sum of polyunsaturated straight-chain fatty acids*	*13.0*
* Total others*	*7.8*

^1^ Only fatty acid methyl esters in quantities greater 0.1% were considered.

## Supplementary Materials

The following are available online at https://www.mdpi.com/2218-273X/10/9/1259/s1, Table S1: Characteristics of gene-specific primers used for qPCR analysis.
