# Supplementary material for: Branched-Chain Fatty Acids as Mediators of the Activation of Hepatic Peroxisome Proliferator-Activated Receptor Alpha by a Fungal Lipid Extract
Source: Biomolecules. 2020 Aug 31;10(9):1259. doi: 10.3390/biom10091259 (PMC7565516; doi:10.3390/biom10091259)
Supplement: Supplementary file 1 [file biomolecules-10-01259-s001.pdf]

Supplementary Materials

Branched-Chain Fatty Acids as Mediators of the Activation of Hepatic Peroxisome Proliferator-Activated Receptor Alpha by a Fungal Lipid Extract

Garima Maheshwari [1,2], Robert Ringseis [1,*], Gaiping Wen [1], Denise K. Gessner [1], Johanna Rost [2], Marco A. Fraatz [2], Holger Zorn [2,3] and Klaus Eder [1]

1 Institute of Animal Nutrition and Nutrition Physiology, Justus Liebig University Giessen, Heinrich-Buff-Ring 26-32, 35392 Giessen, Germany; Garima.Maheshwari@lcb.chemie.uni-giessen.de (G.M.); gaiping.wen@ernaehrung.uni-giessen.de (G.W.); denise.gessner@ernaehrung.uni-giessen.de (D.K.G.); Klaus.Eder@ernaehrung.uni-giessen.de (K.E.)

2 Institute of Food Chemistry and Food Biotechnology, Justus Liebig University Giessen, Heinrich-Buff-Ring 17, 35392 Giessen, Germany; johanna.rost@gmx.de (J.R.); marco.fraatz@lcb.chemie.uni-giessen.de (M.A.F.); holger.zorn@lcb.chemie.uni-giessen.de (H.Z.)

3 Fraunhofer Institute for Molecular Biology and Applied Ecology, Winchester Str. 2, 35394 Giessen, Germany

* Correspondence: robert.ringseis@ernaehrung.uni-giessen.de; Tel.: +49-641-9939231

Received: 8 June 2020; Accepted: 28 August 2020; Published: date

Supplementary Table S1

Characteristics of gene-specific primers used for qPCR analysis.

Gene	Forward Primer (3´-5´)	Reverse Primer (3´-5´)	Product length (bp)	T_m (°C)	NCBI GenBank
Reference genes					
ACTB	GACCTCTATGCCAACACAGT	CACCAATCCACACAGAGTAC	154	60	NM_031144
ATP5B	GCACCGTCAGAACTATTGCT	GAATTCAGGAGCCTCAGCAT	203	60	NM_134364
CANX	CCAGATGCAGATCTGAAGAC	CTGGGTCCTCAATTTCACGT	175	60	NM_172008
MDH1	CAGACAAAGAAGAGGTTGCC	CGTCAGGCAGTTTGTATTGG	206	60	NM_033235

RPL13	CTTAAATTGGCCACGCAGCT	CTTCTCAACGTCTTGCTCTG	198	60	NM_031101
TOP1	GAAGAACGCTATCCAGAAGG	GCTTTGGGACTCAGCTTCAT	137	60	NM_022615
Target genes					
ACOX1	CTGGGCTGAAGGCTTTTACT	GCTGTCTGCAGCATCATAAC	172	60	NM_017340
CPT1A	GCAAAGTGTCGGCAGACCT	CATCTTCCATGCAGCAGGGA	170	60	NM_031559
CYP4A1	CAGAATGGAGAATGGGGACAGC	TGAGAAGGGCAGGAATGAGTGG	460	60	NM_175837
SLC22A5	GAACTCACGAGCCTCGCACGC	TCGTCGTAGTCCCGCATGCC	117	60	NM_019269

Abbreviations: ACTB, actin, beta; ATP5B, ATP synthase, H+ transporting, mitochondrial F1 complex, beta polypeptide; CANX, calnexin; MDH1, malate dehydrogenase 1, NAD (soluble); RPL13, ribosomal protein 13; TOP1, topoisomerase (DNA) I; ACOX1, acyl-CoA oxidase; CPT1A, carnitine-palmitoyltransferase 1A; CYP4A1, cytochrome P450 4A1; SLC22A5, solute carrier family 22 (organic cation/carnitine transporter), member 5
